# The Amende

**Published:** 1886-01

**Authors:** 


### THE AMENDE.

We extracted a report of three remarkable monstrosities last month from that excellent and progressive periodical, the *Atlanta Medical and Surgical Journal*, and by some kind of inadvertence or accident—certainly far from design - credited it to the *St. Louis Courier of Medicine*. We scarcely know how to apologize to our esteemed cotemporary for such a great injustice, especially seeing we had kicked against a similar crime (?) on the part of our St. Louis cotemporary of the *American Medical Journal;* but we *do* apologize, and take this opportunity of assuring our friend Gray and our readers that *not for the world* would we have done such a thing intentionally. It must have been that, reading both journals about the same time, we got them mixed; and deceived, too, by the similarity of the external appearance, we credited the article to the wrong journal. A thousand pardons, friend!

